# Zinc Level and Prevalence of Rejection in Transplanted Patients

**Published:** 2011-08-01

**Authors:** J Roozbeh, M Sharifian Dorche, R Afshariani

**Affiliations:** 1 Urology Nephrology Research Center, Depart-ment of Internal Medicine, Shiraz University of Medical Sciences, Shiraz, Iran; 2 Department of Neurology, Shiraz University of Medical Sciences, Shiraz, Iran; 3 Department of Public Health, School of Health and Nutrition, Shiraz University of Medical Sciences, Shiraz, Iran

**Keywords:** Zinc, Prevalence, Transplant

Dear Editor,

High zinc level by promoting the immune system may cause rejection in the allograft organ. So it seems that limitation of zinc supplement and all foods and drugs with high amount of zinc may be effective in prevention of rejection after transplantation.

Zinc is an essential micronutrient for human growth, development and immune function.

Zinc deficiency was accompanied by the qualities and functional insufficiency of T-cell mediated immunity and by the diminished phagocytic activity of neutrophils.[1]

Kabu et al. in July 2006 revealed that zinc was involved in multiple step of FC epsilon RI-induced mast cell activation and required for degranulation of cytokines such as IL-6 and TNF-α production and lymphocytes proliferation.[1][2]

In study by Chen et al. (Nov 2005) on the effects of different levels of zinc nutrition status on the immune function of mice spleen lymphocytes showed that zinc status affected the immune function and production of IL-2 in spleen lymphocytes [3] Rejection is one of the most important problems after organ transplantation. Immune system has a critical role in this process .In the context of allograft rejection, T cells play a central role in the immune response, once activated, they secrete cytokines and chemokines to activate and attract cells such as CD8 T cells and macrophages into the allograft. They also interact with B cells that secrete alloreactive antibodies that eventually lead to allograft destruction. [4]

Moreover transplant rejection has both cellular and humeral components, some of the cytokines produced by T cells and macrophages (TNF-α) may mediate apoptosis of graft cells. [5] According to all those facts, it could be hypothesized that high zinc level by promoting the immune system may cause rejection in the allograft organ.
